# Systems biology analysis of immune signaling in peripheral blood mononuclear cells (PBMC) of melanoma patients receiving ipilimumab; basis for response biomarker identification

**DOI:** 10.1186/1479-5876-12-S1-O13

**Published:** 2014-05-06

**Authors:** Drew Hotson, Ryan Alvarado, Andy Conroy, Santosh Putta, Ester Simeone, Assunta Esposito, Mariaelena Capone, Gabriele Madonna, Antonio M Grimaldi, David B Rosen, Spencer Liang, Alessandra Cesano, Carmela Cacciapuoti, Paolo A Ascierto, Rachael E Hawtin

**Affiliations:** 1 Nodality, Inc, South San Francisco, CA, USA; 2 G. Pascale National Tumor Foundation Institute, Naples, Italy

## Background

Ipilimumab, an anti-CTLA-4 monoclonal antibody, is approved for treatment of unresectable/metastatic melanoma [1]. Treatment benefits only a subset of patients (pts) and is associated with significant adverse effects. Biomarkers of pt response are needed. Single cell network profiling (SCNP) is a multiparametric flow cytometry-based assay that quantitatively measures both phenotypic markers and changes in intracellular signaling proteins in response to modulation, enabling analysis of cell signaling networks [2,3]. We have been concerned on functional proteomic profiling of immune signaling pathways in PBMC subsets in healthy donors and pts with metastatic melanoma receiving ipilumumab treatment.

## Methods

3 healthy and 13 melanoma pt cryopreserved samples, (7 pre-treatment (pre-Tx), 6 post-treatment (post-Tx)) were analyzed. Cells were assessed for viability, cell subset frequencies and signaling in response to modulation. A node is the combination of modulator and intracellular readout (e.g. IL-6 -> p-STAT3). Cytokine, TCR- or FcγR-induced activation of STAT1, 3, 5, CD3z and Erk was evaluated in immune cell populations and comparisons made based on disease, treatment time point and clinical responses.

## Results

Compared to healthy, melanoma monocytes displayed lower IL-10→p-STAT1, IL-10→p-STAT3, IL-6→p-STAT1, IL-6→p-STAT3 and FcγR→p-Erk. Similarly, TCR→p-CD3 was reduced most prominently in CD45RA- CD4+ and CD4- T cells. These data suggest hyporesponsivity of circulating immune cell subsets in the innate and adaptive arms of the immune response in melanoma pts. The same observations pertained to melanoma samples independent from administration of, or clinical response to, treatment. Effects of treatment on T cell signaling were observed compared to healthy donors and pre-Tx samples. Post-Tx samples had diminished TCR→p-Erk, TCR→p-CD3z in CD4+ and CD4- more prominently in CD45RA+ than CD45RA- subsets and diminished IL-15→p-STAT5 in CD4- T cells. No markers associated with clinical response to ipilumumab. In samples from non-responsive pts, the level of basal p-Erk in CD45RA+ CD4+ T cells and the percentage of Treg was higher compared to those of complete responders and healthy donors.

## Conclusions

SCNP analysis of PBMC from healthy donors and melanoma pts pre-Tx and post-Tx identified signaling differences between melanoma and healthy and between pre- and post-Tx. Candidate predictive biomarkers for patient stratification will be evaluated in subsequent larger studies.
